# Effects of repeated testing in a pen-and-paper test of selective attention (FAIR-2)

**DOI:** 10.1007/s00426-021-01481-x

**Published:** 2021-02-11

**Authors:** Bianca Wühr, Peter Wühr

**Affiliations:** 1 University of Applied Sciences, Dortmund, Germany; 2 Institut Für Psychologie, Technische Universität Dortmund, Emil-Figge-Straße 50, 44227 Dortmund, Germany

## Abstract

The FAIR-2 (‘Frankfurter Aufmerksamkeitsinventar’) is a pen-and-paper test of visual attention in which participants have to search for targets among distractors. For similar pen-and-paper tests of attention (e.g., d2), the repetition of the test causes large improvements in performance that threaten both its (retest) reliability and validity. We investigated the size and possible sources of practice effects in the FAIR-2 in three experiments. In Experiments 1 and 2, participants were tested twice using the original FAIR-2. We compared how performance changed after 2 weeks (Experiment 1) or 3 months (Experiment 2), when the test was repeated (complete repetition), or when targets and distractors changed their roles (test reversal). For Experiment 3, we used self-constructed versions of the FAIR that allowed for a third neutral condition (complete alternation) without any stimulus overlap between the two tests. The complete repetition condition produced strong performance gains (25–35%) that persisted for 3 months. For the complete-alternation condition, we observed small to moderate improvements, suggesting that stimulus-independent learning had occurred in session 1. Finally, performance did not differ between test reversal and complete alternation, therefore, suggesting that improvements in target processing had caused the large improvements in the complete-repetition condition.

**Supplementary Information:**

The online version contains supplementary material available at 10.1007/s00426-021-01481-x.

## Introduction

Working in an attentive and concentrated manner is an important prerequisite for successful behavior in many areas of life, including school and the workplace. To assess a person’s ability to work in an attentive and concentrated manner, psychologists have developed pen-and-paper tests examining selective attention and concentration, such as the d2 test (e.g., Brickenkamp, 1962, 2002) and the ‘Frankfurter Aufmerksamkeitsinventar’ (FAIR; Moosbrugger & Oehlschlägel, 1996). Because the results in such tests may have serious consequences for the tested person, these tests should be both highly reliable and highly valid. However, both the (retest) reliability and the validity of the d2, for example, are curtailed by effects of practice, that is, the fact that repeating the test considerably improves the test results (e.g., Hagemeister & Westhoff, 2011; Schmidt-Atzert, Büttner, & Bühner, 2004). The effects of practice decrease the (retest) reliability of a test because the results of a person in two subsequent tests may differ considerably. Moreover, the effects of practice also threaten the validity of a test because it is unclear whether practice or high ability has caused a good test result in situations where the practice history of the tested person is unknown (Hagemeister & Westhoff, 2011; Schmidt-Atzert et al., 2004).

The FAIR test (Moosbrugger & Oehlschlägel, 1996) was constructed in response to some shortcomings of previous tests, such as the d2 (cf. Oehlschlägel & Moosbrugger, 1991). While both the existence and size of practice effects are empirically documented for the d2 (e.g., Steinborn, Langner, Flehmig, & Huestegge, 2018; review in Hagemeister & Westhoff, 2011), there are no published data on the size and characteristics of practice effects in the FAIR or FAIR-2 (Moosbrugger & Oehlschlägel, 1996,2011). Hence, the main goal of this study is to close this gap and to start exploring the sources of practice effects in the FAIR-2.

### The FAIR-2 test of selective attention

The FAIR-2 (Moosbrugger & Oehlschlägel, 2011) is a tool for the psychometric assessment of selective attention in persons aged between 9 and 72. In particular, the test is thought to assess “focused attention as the ability to quickly and accurately discriminate between visually similar items, and to concurrently ignore task-irrelevant information” (Moosbrugger & Reiß, 2004, p.4, translated by authors). The FAIR-2 has two pages with 16 rows each comprising 20 stimuli. The stimuli were constructed by combining a shape (i.e., circle or square) with two or three dots located inside the shape. The FAIR-2 comes in two versions: In version A, circles with three dots and squares with two dots are targets, whereas circles with two dots and squares with three dots are distractors. Conversely, in version B, targets and distractors are switched. When completing the FAIR-2 test, participants are supposed to draw a continuous line under each stimulus row and to mark each target by drawing an upward spike through the target. This so-called “principle of complete marking” allows the investigator to check the order in which the stimuli were inspected and marked. Participants are given three minutes of time per test page. Performance in the FAIR-2 is described by means of three variables (i.e., *L*, *Q*, and *K*). The *L* measure (from the German word “Leistung”, which means ‘performance’) is the sum of hits and represents the number of correctly detected targets. The *Q* measure (from the German word “Qualität”, which means ‘quality’) is the percentage of correctly inspected items in relation to the number of all inspected items (including errors). Finally, the *K* measure (from the German word “Kontinuität”, which means ‘continuity’) is computed from *L* × *Q* and claims to reflect the stability of performance.

### Practice effects in tests of selective attention

Despite their theoretical and practical relevance, there is a surprising lack of published studies on practice effects in tests of selective attention. With the English version of the d2, Harris, Minassian, and Perry (2007) reported a repetition benefit of 17% (in GZ-F^1^) for adult participants. A somewhat smaller repetition benefit of approximately 11% (in GZ-F) was more recently observed by Steinborn et al. (2018). Besides investigating the effects of repeating the d2 test after one week, these authors also addressed short-term changes of performance within a session. Therefore, the authors compared performance in the first half of the d2 (lines 1–7) with performance in the second half (lines 8–14), observing a decline in the performance of about 3.3% (in GZ-F). These so-called “time-on-task” effects could be attributed to an increase in tiring and/or a decrease in motivation.

### Practice effects in conjunction-search tasks: methods, findings, and accounts

From the viewpoint of cognitive psychology, both the d2 and the FAIR-2 tests require a visual search for conjunction targets that are hidden between heterogenous distractor stimuli (for reviews of the rich literature on visual search, see Chan & Hayward, 2013, and Wolfe, 1998). In conjunction-search tasks, the targets are defined by a particular combination of features, whereas other combinations occur as distractor stimuli. The visual search for conjunction targets among distractors, which share features with the targets, requires the formation of internal target representations—the so-called “search templates” (e.g., Bravo & Farid, 2012; Wolfe & Horowitz, 2004). Simple visual search models assume that the stimulus display is then attentively searched and every stimulus (representation) is compared to the search template(s). Depending on whether this comparison produces a match or a mismatch, a corresponding response is made (e.g., Treisman & Gelade, 1980; Wolfe, 1998).

Research on the impact of practice on visual search has a long tradition, too. For example, in a classic study by Neisser (1963), participants practiced searching for a variable number of targets for up to 31 days. At the beginning of the practice, search times increased with the number of targets being searched for, but these differences decreased with practice and had almost vanished after 31 days of practice. This finding suggests that, after sufficient practice, the processes of visual search may become automatic and, therefore, depend less on limited resources such as attention. According to this notion, whereas participants have to direct their attention to each item in unpracticed searches, the target items may (automatically) attract attention towards their position after extended practice (also see Shiffrin & Schneider, 1977).

Subsequent studies demonstrated that both the type of stimulus material and the stimulus–response mapping affect practice in visual conjunction-search. Concerning the stimulus material, substantial practice effects have been demonstrated when participants searched for arbitrary combinations of line segments (e.g., Czerwinski, Lightfoot, & Shiffrin, 1992; Lubow & Kaplan, 1997), letters (e.g., Neisser, 1963; Prinz, 1979) or words (e.g., Fisk, Lee, & Rogers, 1991; Rogers & Fisk, 1991). Moreover, whereas practice can improve searches for conjunctions of color and location (e.g., Frank et al., 2014), practice does not seem to affect search for conjunctions of color and shape (e.g., Leonards et al., 2002; Sireteanu & Rettenbach, 2000). Additionally, the consistency of the stimulus–response mapping can also affect practice in visual search. In consistent-mapping (CM) conditions, stimulus X is always a target and stimulus Y is always a distractor, whereas in variable-mapping (VM) conditions each stimulus is sometimes a target and sometimes a distractor. Several studies have identified that practicing a search task under CM conditions can produce much bigger improvements in performance than practice under VM conditions (e.g., Fisk et al., 1991; Rogers & Fisk, 1991; Shiffrin & Schneider, 1977). Interestingly, though, it turned out later that the observation of larger practice effects with CM conditions, as compared to VM conditions, seems to be constrained to alphanumerical (i.e., familiar) stimuli, whereas practice can similarly improve performance in both CM and VM conditions with novel stimuli (e.g., Czerwinski et al., 1992; Lightfoot, Czerwinski, & Shiffrin, 1993; Shiffrin & Lightfoot, 1997).

Several accounts for the effects of practice in visual search tasks have been proposed. A first account assumes that practice in visual search tasks mainly changes the attentional “weights” of both the targets and the distractors. In particular, in their “*attention-attraction*” model, Shiffrin and Schneider (1977) assume that CM practice increases the attentional weight (or attention-attraction strength) of targets, and decreases the attentional weights of distractors (also see Czerwinski et al., 1992; Rogers, 1992; Shiffrin, 1988). Among other observations (which will be discussed in due course), this account can explain why CM practice leads to larger improvements in performance than VM practice. In fact, according to Shiffrin and colleagues, larger training effects with CM conditions than with VM conditions are a hallmark of the automatization of visual search (e.g., Czerwinski et al., 1992; Lightfoot et al., 1993).

A second group of accounts assumes that practice in visual search tasks improves the perceptual processing of targets, or the perceptual discrimination between targets and distractors (cf. Goldstone, 1998, for a review). According to one account, practice improves the perceptual processing of targets by stimulating the formation of new processing units—a process called ‘unitization—for previously unpracticed stimulus combinations (Czerwinski et al., 1992; Frank et al., 2014; Lightfoot et al., 1993; Shiffrin & Lightfoot, 1997).

According to the third account of perceptual learning, practice improves the perceptual discrimination of targets and distractors, that is, participants learn to detect and process those features that distinguish between targets and distractors in a given task (e.g., Cousineau & Larochelle, 2004; Duncan & Humphreys, 1989; Fisher, 1982; Rabbitt, 1964). If a participant has to search for the letters E and H among distractors K and Z, a short horizontal line in the middle of the stimulus would be a critical feature that could be used to discriminate between targets and distractors.

A series of studies with words provided evidence for the attention-attraction account of practice in visual search (Fisk et al., 1991; Rogers & Fisk, 1991; Rogers, 1992).^2^ Participants first practiced visual search tasks for 10 days under both CM and VM conditions. The task consisted of searching for a word from a pre-cued semantic category among distractor words. After the practice phase, participants were tested in several tests or transfer conditions. A first notable result was that performance improved more strongly during practice with CM conditions than with VM conditions, and the search was faster in CM conditions than in VM conditions at the end of the training. The results from the test conditions typically showed that the repetition of the target or the distractor from practice to test produced a better performance as compared to a control condition, in which neither the target nor the distractor were repeated. In contrast, when either the target, the distractor, or both (i.e., role reversal) switched their role from practice to test, performance deteriorated compared to the control condition. The pattern of findings is consistent with an attention-attraction account (e.g., Shiffrin & Schneider 1977; Shiffrin, 1988), and inconsistent with a perceptual-discrimination account of practice in visual search (e.g., Cousineau & Larochelle, 2004; Duncan & Humphreys, 1989; Fisher, 1982; Rabbitt, 1964). For example, the strong disruption of performance in the role-reversal condition is compatible with the attention-attraction account because, according to this account, participants would have to search for weak targets (i.e., previous distractors) among strong distractors (i.e., previous targets) in this condition. In contrast, according to the perceptual-discrimination account, reversing the roles of targets and distractors should not significantly impair performance because the learned features for discriminating targets and distractors remain the same.

In another series of studies, Shiffrin and colleagues demonstrated the limits of automatization in visual search and provided evidence for the unitization of stimuli as a result of extended practice (e.g., Czerwinski et al., 1992; Lightfoot et al., 1993; Shiffrin & Lightfoot, 1997). These authors reasoned that the stimulus material used in a search task determines how practice would affect performance. In particular, they assumed that the automatization of visual search would mainly occur when targets and distractors were dissimilar, and when the unitization of stimuli was unlikely to occur. Both of these conditions are met with alphanumeric stimuli. In contrast, with unfamiliar stimuli and high similarity between targets and distractors, the practice could improve performance by developing higher-order units for stimulus processing. Thus, “if subjects initially process stimuli at the feature level and later learn to process them holistically, the number of comparisons required to discriminate targets from distractors would go down considerably as training proceeds” (Czerwinski et al., 1992, p 296). The novel stimuli consisted of a rectangular frame that contained three line segments in different configurations. Participants extensively practiced the visual search for a pre-cued target in displays containing a target (or no target) and several distractors. A (unique) combination of two features distinguished a given target from each distractor in each stimulus set, hence, a conjunction search was required. There were several notable results. First, at the beginning of practice, performance was much worse than usually reported in studies with alphanumeric stimuli, suggesting an effect of different familiarity with stimulus sets. Second, practice improved performance to a similar degree in both CM and VM conditions, suggesting that practice did not automatize visual search under these conditions. Third, although practice strongly improved performance, a closer examination of performance suggested that participants were still serially searching for targets after practice, albeit at a much higher rate than at the beginning of practice. Fourth, when participants were transferred to a condition with new sets (i.e., combinations) of practiced stimuli, which still required a conjunction search, performance did not appear to suffer (e.g., Experiment 3 in Shiffrin & Lightfoot, 1997). Fifth, when participants were transferred to a condition with new sets of practiced stimuli that no longer required a conjunction search, because a single feature distinguished each pair of stimuli, performance did not improve as would be expected if participants were still comparing individual features. In summary, the results of these studies provide evidence for the hypothesis that practicing the search for unfamiliar geometrical stimuli can lead to a unitization of the stimulus representations guiding such searches.

In a more recent study, Frank et al. (2014) investigated the neuroanatomical correlates of practice effects in a visual search task. In their study, participants practiced searching for a conjunction of color and location on eight successive days.^3^ Participants were then probed in several test (or transfer) conditions, including role reversal and a control condition involving a conjunction search for a new set of stimuli.^4^ Functional magnetic resonance imaging was used to measure the brain activity of three participants during both the training and test sessions. On the behavioral level, performance improved during practice and seemed to reach an asymptote after 4–5 days. Moreover, performance almost dropped to pre-training levels in the role-reversal condition, as well as in the control condition. On the neuronal level, the authors observed that practice-induced changes in performance were correlated with increasing activity in visual areas (i.e., V1–V4), but were not correlated with activity in areas related to the control of eye movement (i.e., frontal eye fields, supplementary eye fields, superior colliculi). The latter areas were supposed to measure the effects of practice on the control of attention. To account for their results, Frank et al. (2014) suggested that practice effects in their search task had a perceptual locus (e.g., unitization) rather than an attentional one (e.g., automatization of target detection), supporting the earlier results of Shiffrin and colleagues (e.g., Shiffrin & Lightfoot, 1997).

### Contextual cueing

In addition to changes in the attentional weights of stimuli or changes in the cognitive representations of stimuli, participants might also learn to use contextual cues to improve performance in visual search tasks with practice. For example, in an influential study, Chun and Jiang (1998; see also Chun, 2000) demonstrated that participants can implicitly learn the correlation between the configuration of distractor items and the position of the target. In their Experiment 1, participants searched for a rotated ‘T’ under heterogeneously rotated ‘L’ distractors. Each display contained a target, and participants reported the orientation of the target by pressing a key. Importantly, each block of trials contained a set of “old” displays and a set of “new” displays in random order. The old set of displays consisted of 12 stimulus configurations that were repeated throughout the whole experiment, once per block. The new set of displays consisted of 12 different configurations that were newly generated for each block; these served as a control condition. Thirty blocks of trials were grouped into 6 epochs of 5 blocks each. The results showed that starting with epoch 2, RTs were faster for old displays compared to new displays, demonstrating contextual cueing. Hence, participants were able to quickly learn to locate the target in repeated stimulus configurations. A follow-up experiment revealed that learning the relationship between the spatial configuration of distractors and the target location was important for contextual cueing, whereas distractor identities did not play a role (but see Makovski, 2016 for different findings). Subsequent studies on the locus of the contextual-cueing effect suggested that contextual cueing facilitates the attentional guidance of visual searches, rather than facilitating a response to the target (e.g., Harris & Remington, 2017; see Sisk, Remington, & Jiang, 2019 for review).

While contextual cueing may play a role in the d2 test of attention, it cannot do so in the FAIR-2 test. In the d2 test of attention, each of three stimulus lines is repeated several times to obtain a total of 14 lines. In contrast, in the FAIR-2, stimuli are randomly ordered in each line, and no stimulus line occurs twice in the test. As such, the learning of stimulus configurations (or target locations), in the sense of contextual cueing, is not possible in the FAIR-2 test.

### Implications of findings from research on practice effects in the visual search for pen-and-paper tests of visual attention

The empirical evidence suggests that practice may affect visual search at different stages, including changes in the perceptual processing of target stimuli (i.e., unitization) and changes in the attentional guidance of visual search (cf. Czerwinski et al., 1992; Goldstone, 1998; Lightfoot et al., 1993). Despite these findings, different mechanisms seem to be triggered in different situations. Whereas unitization seems to occur in the search for novel stimuli when the similarity of targets and distractors is high, changes in the attentional attractiveness of stimuli seem to occur for familiar stimuli when the similarity of targets and distractors is low (e.g., Lightfoot et al., 1993). In contrast, transfer studies of practice in visual search tasks produced little evidence for the learning of critical features distinguishing targets from distractors.

At present, one can only speculate about the possible sources of practice effects in pen-and-paper tests of visual attention, such as the FAIR-2. In fact, there are many methodological differences between these tests and the typical search tasks used in the laboratory, and empirical studies on the sources of practice effects in pen-and-paper tests of attention do not exist. The FAIR-2 test, however, involves unfamiliar stimuli and high levels of similarity between targets and distractors, creating conditions under which, according to Shiffrin and Lightfoot (1997), practice may lead to unitization rather than changes of attentional guidance.

Finally, besides altering the processing of stimuli, practicing a visual search task may also cause stimulus-independent learning. Stimulus-independent effects of practice, sometimes called “task learning” (e.g., Frank et al., 2014), might benefit performance in different ways, such as improving familiarity with the test situation (i.e., reducing test anxiety), developing more efficient search strategies and improving the execution of motor responses (e.g., Frank et al., 2014; Rogers, 1992; Sireteanu & Rettenbach, 2000; Wühr, 2019).

### Goals of the present study

In the present study, we investigated the presence and characteristics of practice effects on performance in the FAIR-2 test. In particular, we addressed five different issues in three experiments. It should be noted, however, that (a) laboratory research has not yet settled the issue of what exactly causes practice effects in visual search, (b) we are lacking empirical studies on practice effects in pen-and-paper tests of visual search, and (c) there are a lot of methodological differences between search tasks in the laboratory and pen-and-paper tests. These problems prevent us from making strong predictions for our experiments from the literature.

1. Are there repetition gains in the FAIR-2 and, if so, are these comparable in size to the repetition gains observed for the d2 test? Since the d2 and the FAIR-2 involve similar requirements, and performance in both tests is positively correlated (e.g., Moosbrugger & Oehlschlägel, 1996, 2011), one might expect repetition gains in the FAIR-2 that are comparable to those reported in the literature for the d2 (e.g., Harris et al., 2007; Steinborn et al., 2018). On the other hand, however, the two tests differ in many details and the correlations in performance are only moderate. Hence, repetition gains in the FAIR-2 might be quite different from those reported for the d2.
2. How persistent are practice effects in the FAIR-2? The question concerning the persistence of practice effects is mostly of practical relevance. If practice effects in a test of selective attention disappear after a couple of weeks, then these effects would be rather meaningless to people using these tests for practical purposes. We are not aware of any data on the persistence of practice effects with the d2 or the FAIR tests. Therefore, Experiment 2 investigated whether practice effects in the FAIR-2 persist for 3 months.
3. How does performance change within a single session with the FAIR-2? For the d2 test, Steinborn et al. (2018) observed that performance decreased within a single session by about 3% when comparing the first half of the test to the second half. It is unclear whether similar time-on-task effects occur for the FAIR-2 test. It is possible, however, that the negative effects of increasing tiredness or decreasing motivation on performance are counteracted by the positive effects of learning and practice that would improve performance in a subsequent repetition of the same test. Therefore, the negative effects of being tired or losing motivation might only be visible if learning effects are relatively weak. In contrast, if the effects of practice set in very early and are strong, improvements in performance might already be observed during the first session.
4. How does a reversal between targets and distractors, as compared to a complete repetition of the test, affect performance in the FAIR-2 test? We know from earlier studies that role reversal usually deteriorates performance as compared to a complete repetition (e.g., Frank et al., 2014; Prinz, 1979). The size and direction of performance in the role-reversal condition is nevertheless interesting because it provides information about the relative contributions of stimulus-dependent and stimulus-independent learning in the first session with the FAIR-2; the corresponding rationale is further explained in the introduction to Experiment 1. We further explored this issue with a complete-alternation condition, which is introduced in Experiment 3.
5. Finally, we wanted to make a first attempt to explore the subject of stimulus-dependent learning when completing the FAIR-2. Consequently, we constructed eight new versions of the FAIR test that allowed us not only to assess performance in complete-repetition and role-reversal conditions but also in a complete-alternation condition, in which there was no overlap between the stimuli in two subsequent tests. Comparing the results in the three conditions provides the first clues about those cognitive processes that improve when working on the FAIR-2 for the first time.

## Experiment 1

In Experiment 1, we investigated changes in performance when participants did the FAIR-2 test twice within 2 weeks. This study had three goals. First, we wanted to investigate the size of performance improvements that arise from a repetition of the test. Second, we wanted to investigate changes in performance within a single session with the FAIR-2. Therefore, we assessed and analyzed performance separately for the two pages of the test booklet in each session, rather than summarizing across the two pages. Third, we wanted to investigate how reversing the roles of targets and distractors affects performance in the second session. In contrast to the d2, there are two complementary versions of the FAIR-2 test that can be used to compare the effects of a role reversal to the effects of a test repetition. On the basis of previous studies, we expected that performance in the (second session of the) role-reversal condition would be worse than performance in a complete-repetition condition (e.g., Fisk et al., 1991; Frank et al., 2014; Prinz, 1979). While it is clear that performance would improve in the complete-repetition condition from sessions 1 to 2, predictions concerning changes of performance from session 1 to session 2 in the role-reversal condition are difficult. In fact, depending on (a) the content of stimulus-dependent learning in the first session and (b) the relative contributions of stimulus-dependent and stimulus-independent learning, different changes in performance are possible in the role-reversal condition.

**Fig. 1:**
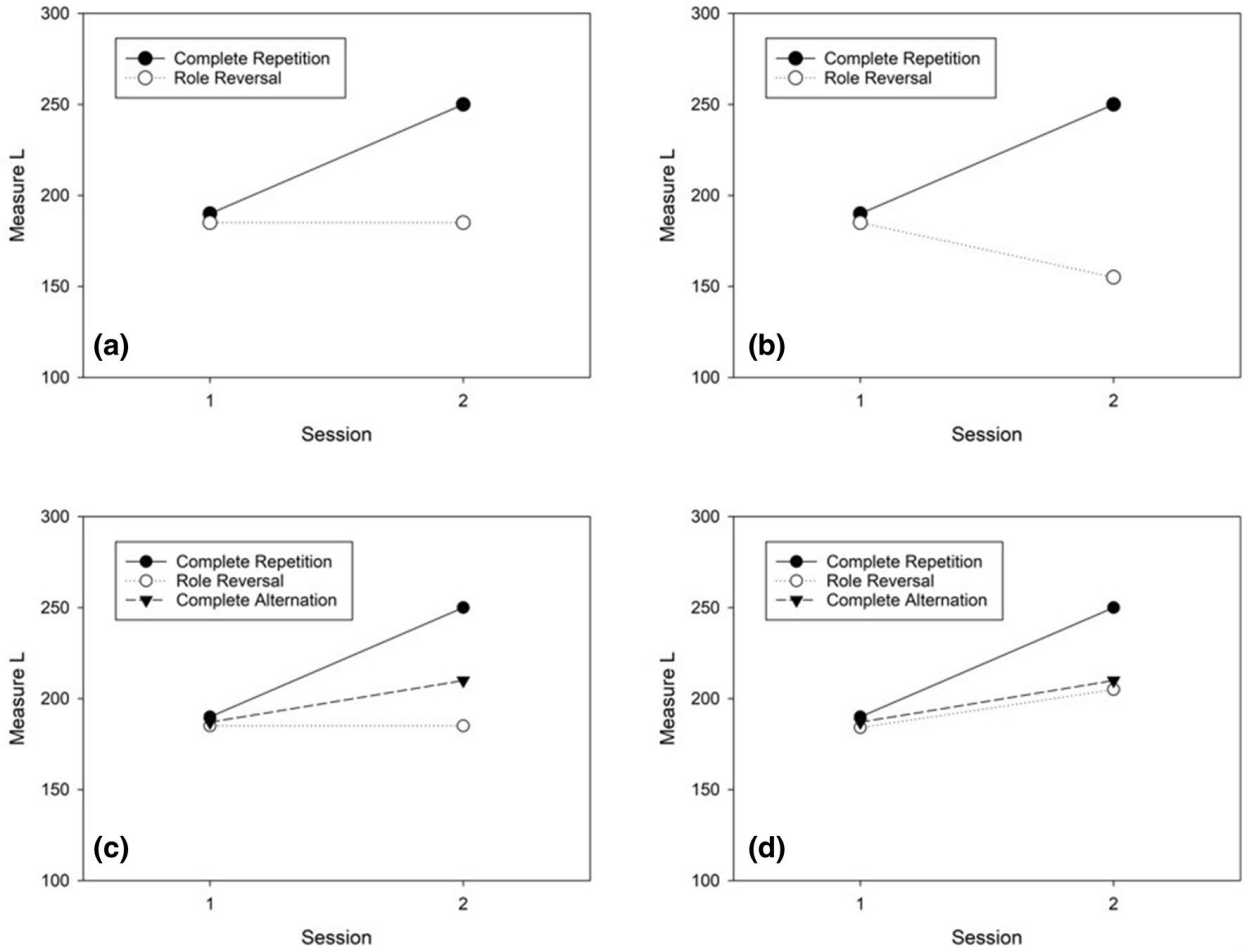
Possible patterns of results in the present experiments (see explanation in the text)

Let us first briefly consider the possible impact of stimulus-dependent learning in the first session on performance in the second session. If practice in the first session only improved the processing of target stimuli (e.g., by unitization; cf. Goldstone, 1998), this learning should improve performance in the second session of the complete-repetition condition, but it should not affect (i.e., neither improve nor impair) performance in the second session of the role-reversal condition (cf. Fig. 1a). If, in contrast, practice in the first session not only improved the processing of targets but also improved the rejection of non-targets, as assumed in the attention-attraction model (e.g., Rogers, 1992; Shiffrin, 1988; Shiffrin & Schneider, 1977), this practice might not only improve performance in the complete-repetition condition but also impair performance in the role-reversal condition (cf. Fig. 1b). In particular, in the second session of the role-reversal condition, performance would suffer from having to search for targets with weak attentional weights among distractors with strong attentional weights.

If stimulus-independent learning occurred in addition to stimulus-dependent learning, then both sources of learning would be expected to improve performance in the second session of the complete-repetition condition (cf. Fig. 1c). Figure 1c also shows the hypothetical performance in a condition, called the “complete-alternation” condition, where two subsequent tests shared only stimulus-independent affordances but did not share stimuli. In the complete-alternation condition, only stimulus-independent learning would occur and improve performance in the second session. In contrast, in the role-reversal condition, stimulus-independent learning from session 1 would improve performance in the second session, whereas stimulus-dependent learning (e.g., having learned to reject stimuli that are now targets) could impair performance in session 2. Hence, if both the positive transfer of stimulus-independent learning and negative transfer of stimulus-dependent learning occurred in the role-reversal condition, and both effects were of similar size (only differing in direction), a pattern as depicted in Fig. 1c could be observed, where performance in the role-reversal condition would not change between sessions. If, however, only the positive transfer of stimulus-independent learning, but no negative transfer of stimulus-dependent learning, occurred in the role-reversal condition, then performance might even improve in the role-reversal condition, albeit not as strongly as in the complete-repetition condition (cf. Fig. 1d).

### Methods

#### Design and data analysis

We planned to analyze the effects of three independent variables, CONDITION (complete repetition vs. role reversal), SESSION (1 or 2) and TEST PAGE (1 or 2) on three dependent variables in separate three-factorial Analyses of Variance (ANOVA). The independent variable CONDITION was varied between participants, whereas the independent variables SESSION and TEST PAGE were varied within participants. The three dependent variables were the L, Q and K measures. The *L* measure is the sum of hits and represents the number of correctly detected targets. The *Q* measure is the percentage of correctly inspected items. Finally, the *K* measure (i.e., the product of *L* × *Q*) claims to reflect the stability of performance. Whilst the usual computation of test scores in the FAIR-2 involves summarizing across both pages of the test, we computed test scores separately for each test page. Besides testing for within-test changes in performance, analyzing the scores separately for each page allows for a more sensitive analysis of practice and transfer effects across sessions because the impact of learning in session 1 may affect performance particularly strongly in the first part of session 2.

ANOVAs and *t* tests are parametric tests that require normally distributed data in each condition. We used Shapiro–Wilk tests to check for the normality of data distributions in each condition before performing omnibus analyses and pair-wise tests. Note, however, that we continued computing ANOVAs as omnibus tests even in the absence of normality (in some or all cells of the design) for two reasons. First, previous studies have shown that the ANOVA is robust against violations of the normality assumption (e.g., Blanca, Alarcón, Arnau, Bono, & Bendayan, 2017; Schmider, Ziegler, Danay, Beyer, & Bühner, 2010). Second, a non-parametric alternative to a multi-factorial ANOVA with mixed designs is not readily available. In contrast, for pair-wise tests, we used *t* tests if the data were normally distributed, and Wilcoxon tests if this was not the case.

We also report an effect-size estimate for each statistical test result. In particular, we report ${\eta }_{\mathrm{p}}^{2}$ as an effect-size measure for omnibus (i.e., *F*) tests, and *d* as an effect-size measure for pair-wise comparisons (i.e., *t* tests or Wilcoxon tests). Note that there are no generally accepted criteria for evaluating an observed effect size. For example, Cohen (1992) suggested *d* = 0.20 as a cut-off value for small effects, *d* = 0.50 as a cut-off for intermediate effects and *d* = 0.80 as a cut-off for large effects. Other authors proposed different criteria, however; for example, Ferguson (2009) recommended *d* = 0.40 as the minimum size representing a “practically” significant effect. According to Rosnow and Rosenthal (2003), psychologists have grown accustomed to referring to *r*s of 0.10, 0.30 and 0.50 as small, moderate and large, respectively. These *r* values correspond to *d* values of 0.21, 0.63, and 1.16. Therefore, we denote effects as small when 0.21 < *d* < 0.63, as moderate when 0.63 < *d* < 1.16, and as large when *d* > 1.16.

#### Participants

With Experiment 1, we were mainly interested in the main effect of SESSION and the two-way interaction between CONDITION and SESSION (repetition vs. role reversal). Previous studies on repetition benefits (using the d2 test) suggested very strong effects.^5^ With G*Power (Faul, Erdfelder, Lang, & Buchner, 2007), we determined that 24 participants per condition would give us sufficient power (i.e., *β* = 0.95) to detect a strong effect (${\eta }_{p}^{2}$ = 0.20), when *α* = 0.05. In Experiment 1, 52 participants took part in the first testing session, and 50 participants returned for the second testing session. Hence, we obtained data from 50 participants aged between 18 and 30 years (*M* = 22.8, SD = 2.8). Most of the participants were women (*N* = 47) and enrolled in psychology or educational science. Participants were randomly assigned to one of the conditions (repetition vs. role-reversal). The two groups were similar in age (*M*_1_ = 23.2, *M*_2_ = 22.4) and had similar ratios of men and women.

#### Materials

We used the regular test booklets of the FAIR-2 (Moosbrugger & Oehlschlägel, 2011) in Experiment 1. We tried to use the two versions of the FAIR-2 (A and B) equally often in both conditions. Consistent with test instructions, participants were asked to draw a continuous line under each stimulus line and to mark each target by making an upward spike through it.

#### Procedure

At the beginning of the first session, participants received a sheet containing information about our study and gave their informed consent to participate. The testing sessions were conducted in lecture halls or seminar rooms at TU Dortmund University. The temporal distance between the two sessions was 14 days for 49 participants, and 16 days for one participant. In the complete-repetition condition, 25 participants were tested twice with the same version of the FAIR-2 (i.e., A–A, B–B). In the role-reversal condition, 25 participants were tested with two different versions of the FAIR-2 (i.e., A–B, B–A).

**Fig. 2:**
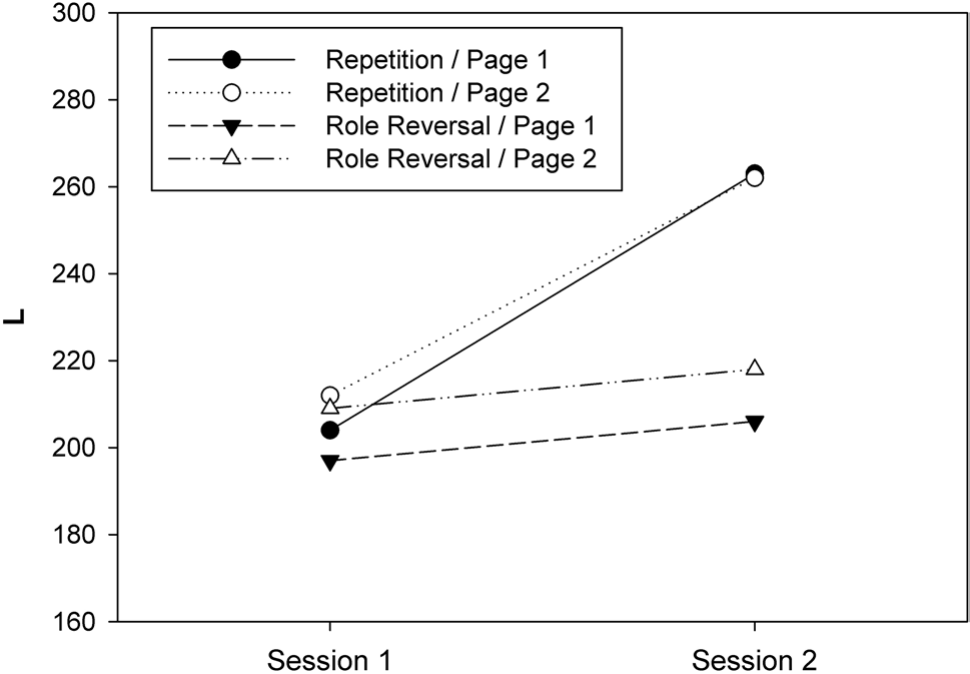
Means of *L* (sum of hits) observed in Experiment 1 as a function of session, test page, and condition

The procedure for the FAIR-2 test was as follows: Participants first filled out their demographic information (e.g., age, gender) listed on the title page. Then participants silently read the instructions for the test (on page 2) and worked through a practice line on page 3. Participants had to read the instructions silently because two different versions of the test (i.e., A and B) were distributed equally among the participants. The tester confirmed that all participants had read and understood the instructions. He or she was equipped with a stopwatch to control the time provided for each page. The tester started the test by saying “Start now!” (in German). After 3 min, he or she said “Stop. Turn the page.” (in German), and participants started working on the second test page. After 3 more minutes, the command “Stop now!” (in German) ended the test (Fig. 2).

### Results

#### Measure *L*

One participant was omitted from the analysis because he/she skipped several lines in one test. Hence, 49 datasets eventually entered into the analysis. Figure 1 shows the means of *L* for the eight cells of our 2 × 2 × 2 design (also see Table 1 in Appendix 1). Shapiro–Wilk tests revealed that the distribution of *L* was consistent with the normality assumption in all cells of the design, all *p*s > 0.25. Next, the individual *L* scores in each cell of the design were subjected to a three-way ANOVA for mixed designs with CONDITION, SESSION and TEST PAGE as independent variables. The numerical results of the ANOVA are presented in Table 2 (cf. Appendix 1). All three main effects were significant. The main effect of CONDITION reflected a higher *L* in the complete-repetition condition (*M* = 235, SD = 48) as compared to the role-reversal condition (*M* = 207, SD = 43). The main effect of SESSION reflected an increase of *L* from the first session (*M* = 205, SD = 40) to the second (*M* = 237, SD = 49). The main effect of TEST PAGE reflected an increase of *L* from the first page (*M* = 217, SD = 48) to the second (*M* = 225, SD = 46).

The most important result was a significant two-way interaction of CONDITION × SESSION (cf. Table 2). To identify the source of the interaction, we compared the *L* scores between the two sessions separately for the two conditions. These comparisons revealed that *L* increased significantly from session 1 (*M* = 208, SD = 38) to session 2 (*M* = 263, SD = 38) in the complete-repetition condition, *t*(23) = 8.48, *p* < 0.001, *d* = 1.730. In contrast, in the role-reversal condition, a numerical increase of *L* from session 1 (*M* = 203, SD = 39) to session 2 (*M* = 212, SD = 44) was not significant, *t*(24) = 1.28, *p* = 0.212, *d* = 0.257. The remaining *F* tests were also not significant (cf. Table 2).

#### Measure *Q*

Table 1 (in Appendix 1) illustrates the means of *Q* for the eight cells of our experimental design. Shapiro–Wilk tests revealed that the distribution of *Q* deviated significantly from normality in seven of the eight cells in our design. We subjected our *Q* data to a three-factorial ANOVA for mixed designs with CONDITION, SESSION and TEST PAGE as independent variables. The numerical results of the ANOVA are presented in Table 3 (cf. Appendix 1). The only significant *F* tests were the main effect of SESSION and the CONDITION × SESSION interaction. The main effect of SESSION reflected an increase of *Q* from session 1 (*M* = 0.947, SD = 0.01) to session 2 (*M* = 0.965, SD = 0.01).

To clarify the source of the two-way interaction, we compared the *Q* scores between the two sessions separately for the two conditions. For the complete-repetition condition, a Wilcoxon test revealed that *Q* significantly increased from session 1 (*M* = 0.938, SD = 0.049) to session 2 (*M* = 0.977, SD = 0.015), *W* = 18.0, *p* < 0.001, *d* = 0.759. In contrast, for the role-reversal condition, the numerical decrease of *Q* from session 1 (*M* = 0.955, SD = 0.023) to session 2 (*M* = 0.942, SD = 0.039) was not significant, *W* = 210.0, *p* = 0.210, *d* = − 0.339. The remaining *F* tests were also not significant (cf. Table 3).

#### Measure *K*

Shapiro–Wilk tests revealed that the distribution of *K* was consistent with the normality assumption for all eight cells in the design (see Table 1 for descriptive statistics). We subjected our *K* data to a three-factorial ANOVA for mixed designs with CONDITION, SESSION and TEST PAGE as independent variables. The numerical results of the ANOVA are presented in Table 4 (cf. Appendix 1). All three main effects were significant. First, a significant main effect of CONDITION reflected higher *K* values for the complete-repetition condition (*M* = 226, SD = 51) than for the role-reversal condition (*M* = 197, SD = 43). Second, a significant main effect of SESSION reflected an increase of *K* from session 1 (*M* = 195, SD = 40) to session 2 (*M* = 229, SD = 42). Third, a significant main effect of TEST PAGE indicated an increase of *K* from page 1 (*M* = 209, SD = 49) to page 2 (*M* = 215, SD = 49).

The only significant interaction was the two-way interaction of CONDITION × SESSION. To clarify the source of this two-way interaction, we compared the *K* scores between the two sessions separately for the two conditions. For the complete-repetition condition, *t* tests revealed that *K* significantly increased from session 1 (*M* = 195, SD = 40) to session 2 (*M* = 257, SD = 39), *t*(23) = 8.517, *p* < 0.001, *d* = 1.739. In contrast, for the role-reversal condition, the numerical increase of *K* from session 1 (*M* = 194, SD = 38) to session 2 (*M* = 201, SD = 45) was not significant, *t*(24) = -0.873, *p* = 0.392, *d* = − 0.175. The remaining *F* tests were also not significant (cf. Table 4).

### Discussion

First, when participants completed the same version of the FAIR-2 test twice, with 2 weeks between the two sessions, performance improved strongly from session 1 to session 2 in the *L* measure (difference = 26%; *d* = 1.73), and performance improved moderately in the *Q* measure (difference = 4%; *d* = 0.76). After 2 weeks, the repetition benefits in the FAIR-2 are, therefore, at least as large as the repetition benefits that previous studies observed for the d2 (cf. Schmidt-Atzert et al., 2004; Hagemeister & Westhoff, 2011; for reviews).

Second, the strong practice benefits that resulted from a repetition of the same test contrast with the effects of a role reversal: When targets and distractors switched between the two sessions, the changes in performance were small (change in *L* = 4%, *d* = 0.26; change in *Q* = − 1%, *d* = − 0.34). Hence, a role reversal between sessions neither improved nor impaired performance in the second session, as compared to the first session (for similar results with different tasks, see Fisk et al., 1991; Prinz, 1979). This pattern of results is consistent with two different interpretations. The absence of a performance change in the role-reversal condition could mean that neither the positive transfer of stimulus-independent learning nor the negative transfer of stimulus-dependent learning occurred in this condition, which would imply that the improvement in the complete-repetition condition fully resulted from the positive transfer of stimulus-dependent learning. Alternatively, the absence of a performance change in the role-reversal condition could also mean that the positive transfer of stimulus-independent learning and the negative transfer of stimulus-dependent learning occurred at similar rates in the role-reversal condition, and their effects cancelled each other out. We will come back to this issue in Experiment 3.

Finally, small improvements in performance could be observed within a session in the *L* measure (difference = 4%, *d* = 0.49), but not in *Q*. The latter observations contrast with those of Steinborn et al. (2018), who observed a decrease in performance within a single session for the d2 test. We will discuss these findings in more detail in the “General Discussion”.

## Experiment 2

The main goal of Experiment 2 was to investigate the persistence of the practice effects in FAIR-2, which we had observed in Experiment 1 with 2 weeks between the sessions. Hence, Experiment 2 is a replication of Experiment 1, but the interval between the two sessions was increased to 13 weeks (i.e., 3 months). If the practice effects, which had occurred after 2 weeks, were no longer visible after 3 months, then the after-effects of a previous encounter with the FAIR-2 would not be meaningful to most practical applications of the test.

### Methods

#### Design and data analysis

The design of Experiment 2 was the same as for Experiment 1. Hence, we planned to analyze the effects of three independent variables, namely CONDITION (complete repetition vs. role reversal), SESSION (1 or 2) and TEST PAGE (1 or 2) on three dependent variables (*L*, *Q*, *K*) in separate three-factorial ANOVAs. The independent variable CONDITION was varied between participants, whilst the independent variables SESSION and TEST PAGE were varied within participants.

#### Participants

We used the effect size of the critical CONDITION × SESSION interaction (${\eta }_{p}^{2}$ = 0.34) from Experiment 1 to calculate the sample size for Experiment 2. To account for the longer interval between the two sessions, we halved the effect size from Experiment 1 to ${\eta }_{p}^{2}$ = 0.17. To detect an effect of this size with high power (i.e., *β* = 0.95; *α* = 0.05), G*Power (Faul et al., 2007) computed a sample size of 34 (per condition). Because we expected a higher dropout rate for Experiment 2, we recruited 40 participants per condition. The 80 participants (67 women, 13 men) in Experiment 2 were aged between 18 and 49 years (*M* = 21.2, SD = 4.5). Participants were randomly assigned to one of the two conditions (repetition vs. role reversal). The two groups were similar in age (*M*_1_ = 20.8, *M*_2_ = 21.5) and had similar ratios of men and women. Fifty-seven participants (71%) completed both testing sessions. Due to this dropout rate, actual power decreased to *β* = 0.91.

#### Materials

We used the regular test booklets of the FAIR-2 (Moosbrugger & Oehlschlägel, 2011) and tried to use both versions equally often in both conditions.

#### Procedure

Again, all participants gave their informed consent to participate at the beginning of the first session. The testing sessions were conducted in lecture halls or seminar rooms at TU Dortmund University. The temporal distance between the two sessions was 91 days for 43 participants and 96 days for 14 participants. In the complete-repetition condition, 27 participants were tested twice with the same version of the FAIR-2 (i.e., A–A, B–B). In the role-reversal condition, 30 participants were tested with two different versions of the FAIR-2 (i.e., A–B, B–A). The testing sessions were conducted as prescribed in the manual.

### Results

#### Measure *L*

**Fig. 3:**
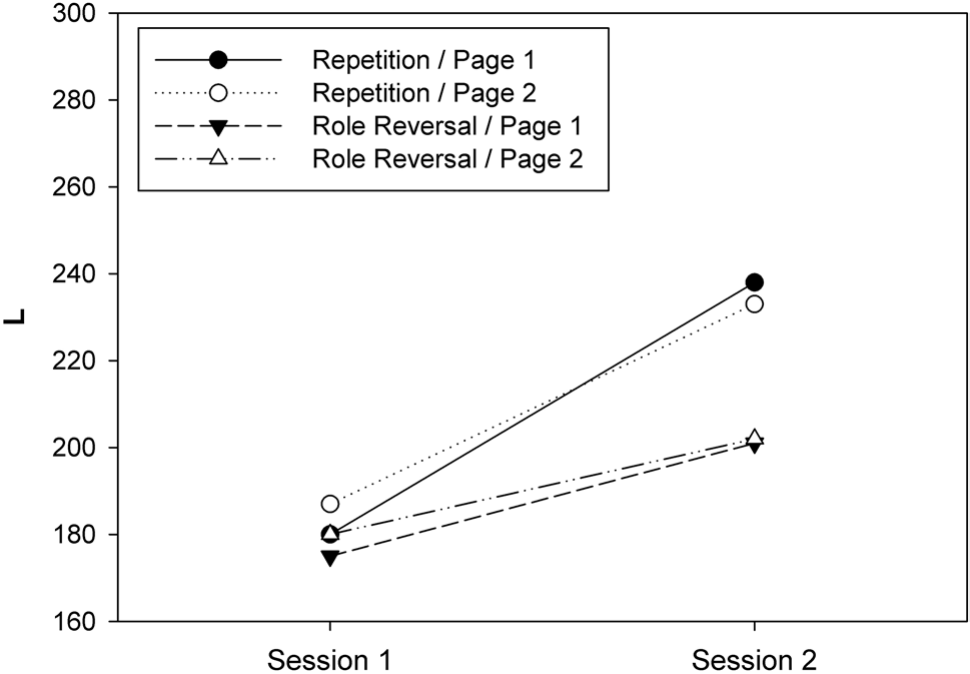
Means of *L* (sum of hits) observed in Experiment 2 as a function of session, test page, and condition

Figure 3 shows the means of *L* for the eight cells of our 2 × 2 × 2 design (also see Table 5 in Appendix 1). Shapiro–Wilk tests demonstrated that the distribution of *L* was consistent with the normality assumption in all cells of our design, all *p*s > 0.18. Next, the individual *L* scores in each cell of the design were subjected to a three-way ANOVA for mixed designs with CONDITION, SESSION and TEST PAGE as independent variables. The numerical results of the ANOVA are presented in Table 6 (in Appendix 1). The main effects of CONDITION and SESSION were significant. The main effect of CONDITION reflected a higher *L* in the complete-repetition condition (*M* = 209, SD = 51) compared to the role-reversal condition (*M* = 189, SD = 38). The main effect of SESSION reflected an increase of *L* from the first session (*M* = 180, SD = 36) to the second (*M* = 217, SD = 45).

Again, the most important result was a significant two-way interaction of CONDITION × SESSION. To identify the source of this interaction, we compared the *L* scores between the two sessions separately for the two conditions; these comparisons revealed that *L* increased significantly between sessions for both the complete-repetition condition (*M*_1_ = 184, *M*_2_ = 235), *t*(26) = 9.86, *p* < 0.001, *d* = 1.900, and the role-reversal condition (*M*_1_ = 177, *M*_2_ = 201), *t*(29) = 4.23, *p* < 0.001, *d* = 0.772. Consequently, the interaction resulted from the fact that *L* increased more strongly in the complete-repetition condition (difference = 51) than in the role-reversal condition (difference = 24).

There was also a significant two-way interaction of SESSION × TEST PAGE. Follow-up tests revealed that *L* increased significantly in session 1 from page 1 (*M* = 177, SD = 36) to page 2 (*M* = 187, SD = 39), *t*(56) = 2.068 *p* = 0.043, *d* = 0.274. In contrast, *L* did not change from test page 1 (*M* = 218, SD = 47) to page 2 (*M* = 216, SD = 43) in session 2, *t*(56) = − 0.800 *p* = 0.427, *d* = − 0.106. The remaining *F* tests were not significant (cf. Table 6).

#### Measure *Q*

Table 5 (in Appendix 1) shows the means of *Q* for the eight cells of our experimental design. Shapiro–Wilk tests revealed that the distribution of *Q* deviated significantly from normal in seven of the eight cells in our design. Despite this finding, we subjected the *Q* data to a three-factorial ANOVA. The numerical results of the ANOVA are presented in Table 7 (in Appendix 1). A significant main effect of CONDITION reflected a higher *Q* score in the complete-repetition condition (*M* = 0.960, SD = 0.029) than in the role-reversal condition (*M* = 0.943, SD = 0.314). A significant main effect of TEST PAGE reflected a decrease of *Q* from page 1 (*M* = 0.958, SD = 0.030) to page 2 (*M* = 0.946, SD = 0.036). The main effect of SESSION was not significant.

The only significant interaction was the two-way interaction of CONDITION × SESSION. To clarify the source of the two-way interaction, we compared the *Q* scores between the two sessions separately for the two conditions. For the complete-repetition condition, a Wilcoxon test revealed that the numerical increase of *Q* from sessions 1 (*M* = 0.955, SD = 0.041) to 2 (*M* = 0.967, SD = 0.022) was not significant, *W* = 124, *p* = 0.121, *d* = 0.355. Similarly, for the role-reversal condition, a Wilcoxon test showed that the numerical decrease of *Q* from session 1 (*M* = 0.950, SD = 0.031) to session 2 (*M* = 0.937, SD = 0.041) was not significant, *W* = 321, *p* = 0.070, *d* = − 0.361. The *F* tests for the remaining interactions were not significant.

#### Measure *K*

Shapiro–Wilk tests revealed that the distribution of *K* was consistent with the normality assumption for all cells in the design (see Table 5 in Appendix 1 for descriptive statistics). We subjected our *K* data to a three-factorial ANOVA for mixed designs with CONDITION, SESSION and TEST PAGE as independent variables. The numerical results of the ANOVA are presented in Table 8 (in Appendix 1). A significant main effect of CONDITION reflected higher *K* values for the complete-repetition condition (*M* = 201, SD = 50) than for the role-reversal condition (*M* = 178, SD = 38). A significant main effect of SESSION reflected an increase of *K* from session 1 (*M* = 172, SD = 38) to session 2 (*M* = 208, SD = 46). The main effect of TEST PAGE was not significant.

There were significant two-way interactions for CONDITION × SESSION, as well as for SESSION x TEST PAGE. To clarify the source of the significant CONDITION × SESSION interaction, we compared the *K* scores between the two sessions separately for each condition. For the complete-repetition condition, *K* significantly increased from session 1 (*M* = 176, SD = 43) to session 2 (*M* = 228, SD = 41), *t*(26) = 9.346, *p* < 0.001, *d* = 1.799. Moreover, *K* also increased significantly from session 1 (*M* = 169, SD = 27) to session 2 (*M* = 189, SD = 41) for the role-reversal condition, *t*(29) = 3.317, *p* = 0.002, *d* = 0.606. Hence, the significant interaction reflected the fact that the increase in *K* from session 1 to session 2 was larger in the former condition than in the latter.

To clarify the source of the significant SESSION × TEST PAGE interaction, we compared the *K* scores between the two pages separately for each session. However, neither the small increase of *K* from page 1 to page 2 in the first session, *t*(56) = 1.290, *p* = 0.202, *d* = 0.171, nor the small decrease of *K* from page 1 to page 2 in the second session, *t*(56) = − 1.634, *p* = 0.108, *d* = − 0.216, was significant. Hence, the significant interaction resulted from the fact that changes in *K* within sessions went in opposite directions. The remaining *F* tests were not significant.

## Discussion

The most important result of Experiment 2 was that the pattern of performance changes between two administrations of the FAIR-2, which had been observed when the two tests were held 2 weeks apart in Experiment 1, still occurred when the two tests were separated by 3 months. A repetition of the test again produced a strong performance improvement in *L* (change = 28%, *d* = 1.90), and a small improvement in *Q* (change = 1.2%, *d* = 0.36). Hence, a repetition of the FAIR-2 seems to produce quite persistent improvements in performance. Furthermore, the large performance improvements in the complete-repetition condition again contrasted with the role-reversal condition: Here, we observed a moderate improvement from session 1 to session 2 in *L* (change = 14%, *d* = 0.77), whereas *Q* remained relatively constant across sessions (change = − 1.4%, *d* = − 0.36). The moderate improvements seen in the role-reversal condition suggest that, after 3 months, the positive effects of stimulus-independent learning in the first session had a stronger impact on performance in the second session than the negative effects of stimulus-dependent learning in the first session.

## Experiment 3

For Experiment 3, we developed eight new versions of the FAIR-2 instead of using the regular test. In contrast to the regular versions of the test, in which each feature dimension (shape, number) has two values, in our new test versions each of the two feature dimensions had four values (cf. Table 9 in Appendix 1). Increasing the number of feature values allowed for the establishment of a new “neutral” condition (i.e., the complete-alternation condition), in which two consecutive tests had no stimulus features in common, in addition to a complete-repetition and a role-reversal condition.

With the new test materials, we pursued two goals in Experiment 3. First, Experiments 1 and 2 had only provided indirect evidence for the notion that stimulus-independent learning in session 1 can improve performance in session 2, and we wanted to test this notion more directly in Experiment 3. Accordingly, we predicted a significant improvement from session 1 to session 2 in the complete-alternation condition.

Second, we made a first attempt to investigate the subject of stimulus-dependent learning in the FAIR-2. As previously described, stimulus-dependent learning in the FAIR-2 might concern (a) the selective improvement of target processing (e.g., by unitization; Czerwinski et al., 1992; Frank et al., 2014), (b) an improvement in the ability to discriminate between targets and distractors by learning distinctive features (e.g., Duncan & Humphreys, 1989; Fisher, 1982; Rabbitt, 1964) or (c) an improvement in the targets’ ability to attract attention and the distractors’ ability to repel attention (e.g., Rogers, 1992; Shiffrin, 1988; Shiffrin & Schneider, 1977). Given the stimulus material in the FAIR-2, however, we consider the possibility of learning distinctive features between targets and distractors to be highly unlikely or even impossible. Note that, in contrast to the stimulus materials used in other tests (e.g., d2) or investigations (e.g., Prinz, 1979; Rogers, 1992; Shiffrin & Schneider, 1977), the targets and distractors of the FAIR-2 consist of different combinations of the same stimulus features. Hence, in the FAIR-2, there is not a single stimulus feature that would allow participants to distinguish between targets and distractors, but it is always the particular combination of features that defines a target or a distractor. Therefore, we assume that stimulus-dependent learning in the FAIR-2 test can only involve the specific improvement of target processing or changes in the attention-attraction values of targets and distractors.

If stimulus-dependent learning in the FAIR-2 mainly increases targets’ ability to attract attention and distractors’ ability to repel attention, the complete-repetition condition should lead to better performance than other conditions. In addition, however, performance in (the second session of) the role-reversal condition should be worse than performance in (the second session of) the complete-alternation condition because targets and distractors switch roles in the role-reversal condition, whereas new targets and distractors with intermediate attention-attraction strengths are presented in the complete-alternation condition (cf. Fig. 1c). Alternatively, if stimulus-dependent learning in the FAIR-2 mainly improves target processing (e.g., through unitization), the complete-repetition condition should lead to better performance than all other conditions. In addition, performance should not differ in (the second sessions of) the role-reversal and complete-alternation conditions because new target stimuli have to be learned in both of these conditions (cf. Fig. 1d).

### Methods

#### Design and data analysis

For Experiment 3, we planned to analyze the impact of three independent variables, CONDITION (complete repetition, complete alternation, role reversal), SESSION (1 or 2) and TEST PAGE (1 or 2) on three dependent variables (*L*, *Q* and *K*) in separate three-factorial ANOVAs. The independent variable CONDITION was varied between participants, whilst the independent variables TEST PAGE and SESSION were varied within participants. In contrast to Experiments 1 and 2, the CONDITION factor had three levels instead of two.

#### Participants

Similar to Experiment 1, we opted a priori for a sample size of 24 participants per condition in Experiment 3. This sample size gives the experiment sufficient power (i.e., *β* = 0.95) to detect a strong effect (${\eta }_{p}^{2}$ = 0.20), with *α* = 0.05. Hence, the complete sample for Experiment 3 comprised 72 participants (60 women, 12 men), aged between 18 and 36 years (*M* = 21.4, SD = 3.6). Participants were randomly assigned to one of the three conditions. The three groups were similar in age (*M*_1_ = 21.4, *M*_2_ = 20.1, *M*_3_ = 23.2) and had similar ratios of men and women. Sixty-nine participants (96%) completed both testing sessions.

#### Materials

We constructed eight new versions of the FAIR test for Experiment 3.^6^ The stimuli were built from different combinations of the two feature dimensions ‘shape’ and ‘number of dots’. The stimulus shape could be a circle, a square, a diamond or a triangle; the number of dots inside the shape could vary between 1 and 4. Table 9 (in Appendix 1) provides information about the stimulus configurations in the eight versions of the new test. We did not use all possible combinations of shapes and dots, but took care to ensure that each of the 16 possible combinations of shapes and dots occurred once as a target and once as a distractor. In every other respect, the test booklets were as similar as possible to the booklets of the FAIR-2 test. Hence, each booklet contained a title page, two pages with instructions and two test pages with 16 lines of 20 stimuli each. In each line, each of the four possible stimuli in a test were presented five times in random order. The size and the spatial arrangement of stimuli most closely resembled those of the FAIR-2.

#### Procedure

Again, all participants gave their written informed consent to participate at the beginning of the first session. The testing sessions were conducted in lecture halls or seminar rooms at TU Dortmund University. The temporal distance between the two sessions was 8 days (i.e., one week). In the complete-repetition condition, 24 participants were tested twice with the same test (e.g., 1A–1A). In the complete-alternation condition, 23 participants were tested with two tests that contained different sets of targets and distractors. Hence, in the complete-alternation condition, four pairs of tests (1A/4A, 1B/4B, 2A/3A, 2B/3B) were administered in two possible orders. Finally, in the role-reversal condition, 22 participants were tested using two different tests in which targets and distractors were switched. Hence, in the role-reversal condition, four new pairs of tests (1A/1B, 2A/2B, 3A/3B, 4A/4B) were administered in two possible orders. Otherwise, the testing sessions were conducted as instructed in the manual.

### Results

#### Measure *L*

**Fig. 4:**
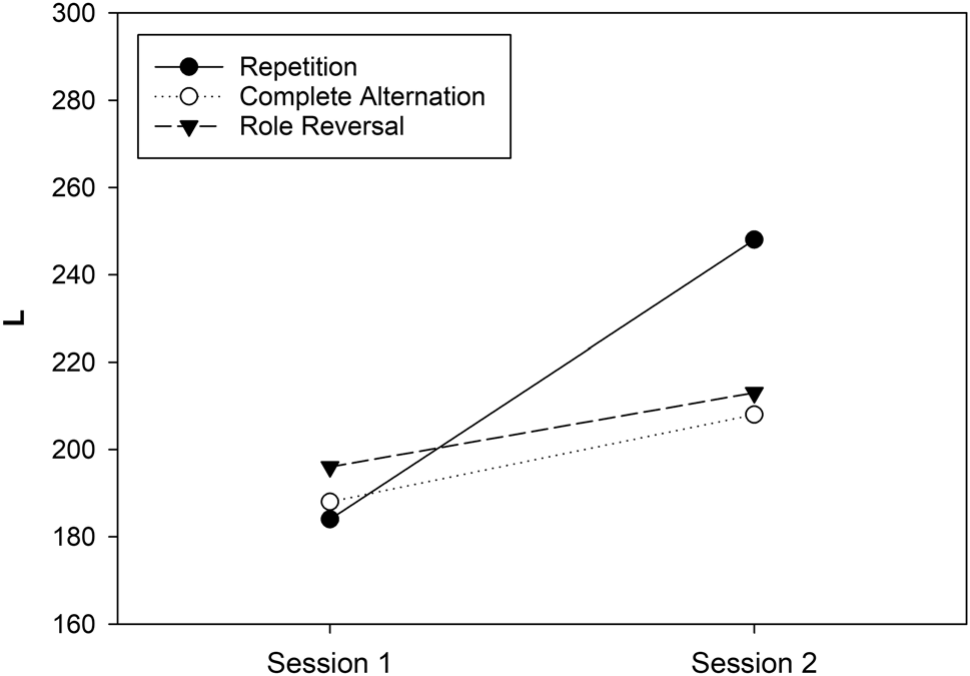
Means of *L* (sum of hits) observed in Experiment 3 as a function of session, test page, and condition

Due to many line errors (i.e., violations of the instruction of drawing a continuous line), the results of one participant were excluded from the data analysis. Thus, the sample was reduced to *N* = 68 (*N*_1_ = 24, *N*_2_ = 23, *N*_3_ = 21). Table 10 (in Appendix 1) shows the means of *L*, *Q* and *K* for all cells in the three-factorial design. Figure 4 shows the means of *L* in all combinations of two sessions and three conditions, averaged across test pages because this variable did not have any significant effect in Experiment 3. Shapiro–Wilk tests determined that the distribution of *L* was consistent with the normality assumption in all cells of the design, all *p*s > 0.06. Next, the individual *L* scores in each cell in the design were subjected to a three-way ANOVA for mixed designs with CONDITION, SESSION, and TEST PAGE as independent variables. The numerical results of the ANOVA are presented in Table 11 (in Appendix 1). A significant main effect of SESSION indicated an increase in *L* from session 1 (*M* = 190, SD = 38) to session 2 (*M* = 223, SD = 45).

A second significant finding concerned the two-way interaction of CONDITION × SESSION. The increase in *L* was much larger in the complete-repetition condition (*M*_difference_ = 65, SD = 22) compared to the complete-alternation condition (*M*_difference_ = 20, SD = 27) and the role-reversal condition (*M*_difference_ = 16, *SD* = 41), with the two latter conditions not differing very much. The improvements in performance were statistically different from zero for both the complete-repetition condition, *t*(23) = 14.48, *p* < 0.001, *d* = 2.956, and the complete-alternation condition: *t*(22) = 3.453, *p* = 0.002, *d* = 0.720. In contrast, the numerical improvement in the role-reversal condition failed to achieve significance, *t*(20) = 1.784, *p* = 0.090, *d* = 0.389. Pair-wise comparisons further showed that the improvement in the complete-repetition condition was larger than changes in the complete-alternation condition, *t*(45) = 6.270, *p* < 0.001, *d* = 1.830, and the role-reversal condition, *t*(43) = 5.040, *p* < 0.001, *d* = 1.507. In contrast, however, changes in the complete-alternation condition and the role-reversal condition did not differ, *t*(42) = 0.344, *p* = 0.733, *d* = 0.104.

#### Measure *Q*

We subjected the means of *Q* (cf. Table 10 in Appendix 1) to a three-factorial ANOVA, with CONDITION, SESSION and TEST PAGE serving as independent variables, although Shapiro–Wilk tests had revealed that the distribution of *Q* was not normal in most cells of the design. The numerical results of the ANOVA are presented in Table 12 (in Appendix 1). The only significant effect was the two-way interaction of CONDITION × SESSION. Indeed, *Q* increased from the first to the second session in the complete-repetition condition (*M*_difference_ = 0.027, SD = 0.043), but decreased in both the complete-alternation condition (*M*_difference_ = − 0.001, SD = 0.026) and in the role-reversal condition (*M*_difference_ = − 0.005, SD = 0.064). In fact, only the improvement in the complete-repetition condition was significantly different from zero, *t*(23) = 3.01, *p* = 0.006, *d* = 0.615. The small decreases in performance in the complete-alternation condition, *t*(22) = − 0.206, *p* = 0.839, *d* = − 0.043 and the role-reversal condition, *t*(20) = − 0.344, *p* = 0.735, *d* = − 0.075, were not significantly different from zero. In addition, pair-wise comparisons showed that the improvement in the complete-repetition condition was significantly larger than the change observed in the complete-alternation condition, *t*(45) = 2.660, *p* < 0.011, *d* = 0.776, but only numerically larger than the change observed in the role-reversal condition, *t*(43) = 1.95, *p* = 0.058, *d* = 0.583.

#### Measure *K*

Shapiro–Wilk tests revealed that the distribution of *K* was consistent with the normality assumption for ten of the twelve design cells. The means are shown in Table 10 (Appendix 1). We subjected our *K* data to a three-factorial ANOVA for mixed designs with CONDITION, SESSION and TEST PAGE as independent variables. The numerical results of the ANOVA are presented in Table 13 (in Appendix 1). The only significant main effect of SESSION reflected an increase of *K* from session 1 (*M* = 180, SD = 37) to session 2 (*M* = 215, SD = 47).

The only significant interaction was the CONDITION × SESSION interaction. To clarify the source thereof, we compared the *K* scores between the two sessions separately for each condition. In numerical terms, the increase in *K* was much larger in the complete-repetition condition (*M*_difference_ = 68, SD = 24) compared to the complete-alternation condition (*M*_difference_ = 19, SD = 26), as well as the role-reversal condition (*M*_difference_ = 16, SD = 45), with the latter two conditions being broadly similar. The improvements in performance were statistically different from zero for the complete-repetition condition, *t*(23) = 13.671, *p* < 0.001, *d* = 2.791, and the complete-alternation condition, *t*(22) = 3.450, *p* = 0.002, *d* = 0.719. In contrast, the numerical improvement in the role-reversal condition missed significance, *t*(20) = 1.649, *p* = 0.115, *d* = 0.360. Pair-wise comparisons further showed that the improvement in the complete-repetition condition was larger than the changes in the complete-alternation condition, *t*(45) = 6.715, *p* < 0.001, *d* = 1.959, and the role-reversal condition, *t*(43) = 4.882, *p* < 0.001, *d* = 1.459. In contrast, changes in the complete-alternation condition and the role-reversal condition did not differ, *t*(42) = 0.226, *p* = 0.822, *d* = 0.068. The remaining *F* tests were not significant.

### Discussion

In Experiment 3, we used eight self-constructed versions of the FAIR-2 test that allowed the creation of a complete-alternation condition, which did not involve overlap between the stimuli in two tests, in addition to a complete-repetition condition and a role-reversal condition. Experiment 3 produced three notable results. First, with regard to the findings of Experiment 1, we replicated the strong performance improvement in the complete-repetition condition in the *L* measure (change = 36%, *d* = 2.96), while performance in the role-reversal condition showed only a small numerical improvement (change = 9%, *d* = 0.39). Second, in the complete-alternation condition, we observed a moderate improvement of performance from the first to the second session in the *L* measure (change = 10%, *d* = 0.72). This observation provides (novel) direct evidence for the notion that stimulus-independent learning can occur in the (first session of a) FAIR test. Third, when compared directly, the role-reversal condition and the complete-alternation condition showed similar changes in performance from the first to the second session. This finding suggests that stimulus-dependent learning in the first session with the FAIR test mainly improved the processing of target stimuli.

## General discussion

The main purpose of the present study was to investigate the effects of repeated testing with the FAIR-2 test, a pen-and-paper test of selective visual attention. In the following sections, we summarize our main findings and discuss a number of theoretical and practical implications thereof.

### Test repetition effects in the FAIR-2

A single previous testing with the FAIR-2 produces large performance benefits in measures of search speed (i.e., *L*), and small benefits in search accuracy (i.e., *Q*), when the test is repeated. In particular, concerning search speed (*L*), we observed large repetition benefits in Experiments 1 (change = 26%, *d* = 1.83) and 2 (change = 28%, *d* = 1.90). Concerning search accuracy (*Q*), we observed a moderate repetition benefit in Experiment 1 (change = 4%, *d* = 0.76) and a small numerical benefit in Experiment 2 (change = 1%, *d* = 0.36). Given the fact that repetition benefits have previously been reported for other pen-and-paper tests of selective attention (e.g., d2, Steinborn et al., 2018), observing these practice effects from a single previous encounter in the FAIR-2 may be unsurprising; however, this is the first empirical report on the size and characteristics of these repetition benefits. When compared to the d2 test, for which repetition benefits between 10 and 20% were reported in search speed (i.e., in the GZ-F score; Steinborn et al., 2018), the repetition benefits in the FAIR-2 may be even larger. A possible explanation for these larger benefits may be related to the fact that this test involves less stimuli to learn than the d2.

The fact that test repetitions improve both the speed and the accuracy of search performance precludes an interpretation of these in terms of changes in speed-accuracy trade-offs between sessions. Instead, the results suggest that, in the first testing session, the participants acquire and improve capabilities that facilitate search performance in the second session. In particular, our results (as described in more detail below) suggest that participants acquire and improve both stimulus-dependent and stimulus-independent search capabilities (also see Wühr, 2019).

### Stimulus-independent learning in the FAIR-2

Performing a pen-and-paper test of visual attention, such as the FAIR-2, not only requires the processing of targets and distractors but also stimulus-independent processing such as, for example, controlling the motor processes required to follow the stimulus line and mark a target (e.g., Frank et al., 2014; Rogers, 1992; Sireteanu & Rettenbach, 2000; Wühr, 2019). Hence, it seems fairly likely that when performing the test (for the first time) participants learn and improve these stimulus-independent processes, and this learning should benefit performance in subsequent encounters with tests requiring the same stimulus-independent processes. The improvement in performance that would occur in a “neutral” test condition, in which two subsequent tests are similar except for targets and distractors, should provide an estimate of the size of stimulus-independent learning.

In Experiment 3, we established a “neutral” or complete-alternation condition between two sessions with our self-constructed versions of the FAIR test. The results showed a moderate improvement in search speed (change in *L* = 20, *d* = 0.720), whereas search accuracy remained the same (change in *Q* = − 0.10%, *d* = − 0.043). The results disproved the hypothesis of symmetrical transfer effects of stimulus-dependent learning in the complete-repetition and role-reversal conditions because performance (changes) in the complete-alternation condition was not in between the performance changes observed in the former conditions. Rather, performance (changes) in the complete-alternation condition were not significantly different from performance (changes) in the role-reversal condition.

The subjects of stimulus-independent learning may involve somewhat unspecific things such as becoming familiarized with the test situation, or more specific things such as controlling the motor processes required to follow the stimulus line and mark targets in the FAIR-2. Subsequent studies could investigate the role of practicing motor-related requirements by comparing a condition in which the way of marking targets is repeated to a condition in which the way of marking targets is alternated.

### Stimulus-dependent learning in the FAIR-2

In Experiment 3, we attempted to explore the subject of stimulus-dependent learning in the FAIR-2 for the first time. This kind of learning might concern (a) the selective improvement of target processing (e.g., by unitization; Czerwinski et al., 1992; Frank et al., 2014), (b) an improvement of the ability to discriminate between targets and distractors by learning distinctive features (e.g., Cousineau & Larochelle, 2004; Duncan & Humphreys, 1989; Fisher, 1982; Rabbitt, 1964) or (c) changes in the attention-attraction strengths of targets and distractors (e.g., Rogers, 1992; Shiffrin, 1988; Shiffrin & Schneider, 1977). Due to specific characteristics of the stimulus material in the FAIR-2, we precluded the learning of distinctive features identifying targets from distractors in this test.

To assess the remaining possibilities, we compared performance changes in three conditions, namely, a complete repetition of the test, a complete alternation of targets and distractors, and a role reversal between targets and distractors. The results showed a large performance improvement (from the first to the second session) in the complete-repetition condition (35% in *L*, *d* = 2.96), a moderate improvement in the complete-alternation condition (10% in *L*, *d* = 0.72) and only a numerical improvement in the role-reversal condition (9% in *L*, *d* = 0.39). It should be highlighted that the performance improvement in the complete-repetition condition was larger than in the remaining conditions, whereas changes in the complete-alternation and role-reversal conditions did not differ. A similar pattern of findings has previously been observed (e.g., Fisk et al., 1991; Prinz, 1979), albeit with words and letters as stimuli.

The attention-attraction model (e.g., Rogers, 1992; Shiffrin, 1988) cannot explain the observed pattern of findings. This model predicts worse performance in the second session with the role-reversal condition, as compared to the second session of the complete-alternation condition, because the learned pattern of attention-attraction values is inverted in the role-reversal condition, and should impair performance in the second session of this condition. Despite this prediction, such a pattern was not observed.

The best account for this pattern of findings assumes that participants mainly learned and improved the processing of targets in the first session (e.g., Czerwinski et al., 1992; Frank et al., 2014). Target-specific learning should improve performance in the complete-repetition condition because participants again search for the “old” (i.e., learned) targets. Conversely, target learning in the first session should not notably affect performance in the remaining conditions because participants have to search for new targets in both conditions.

### Persistence of learning effects

Our experiments demonstrate that the consequences of completing the FAIR-2 once do not disappear within a few days or even weeks. Rather, the consequences of a single testing can persist for at least 3 months without obvious decay. In fact, the pattern of performance changes observed after 3 months did not (statistically) differ from the pattern of performance changes observed after 2 weeks (cf. Appendix 2). The persistence of practice effects in the FAIR-2 has implications for research and practice. An obvious implication for research on learning in visual search is the insight that practice effects, which are often investigated and observed for only very short temporal intervals in the laboratory (e.g., Cousineau & Larochelle, 2004; Prinz, 1979; Shiffrin & Schneider, 1977), may persist over very long intervals. The FAIR-2 test seems particularly suited to investigate the persistence of practice effects in visual search because it involves artificial stimuli, which participants will not encounter outside the laboratory, and therefore any additional experience with the stimulus material of the FAIR-2 between two testing sessions is very unlikely. A clear implication for practical purposes is that practitioners should not simply ignore them. If practice effects in the FAIR-2 quickly disappeared, that is, within a few days or a couple of weeks, their impact on test results outside the laboratory could be ignored because it is highly unlikely that the same person is tested twice (by different persons) in such short periods of time. The present results suggest, however, that practice effects in the FAIR-2 persist for several months (without much decay), and we cannot exclude the possibility that they could even persist for a year or more. Obviously, the longer the practice effects persist, the higher the probability that previous encounters with the FAIR-2 will affect the results of subsequent testing.

### Changes in performance within a session

Besides investigating the changes in performance between two testing sessions, we also addressed the changes in performance within a session with the FAIR-2. Therefore, we compared performance in the first half of the test (i.e., for the first test page) with performance in the second half (i.e., for the second test page). These comparisons revealed rather mixed results, however. In Experiment 1 (2 weeks), we observed a small improvement in *L* (change = 4%, *d* = 0.49) from the first to the second test page, whereas *Q* remained constant within a session. In Experiment 2 (3 months), we observed small decrements in *L* (change = − 4%, *d* = 0.27) during the first session only, whereas *Q* decreased (change = − 1.3%, *d* = 0.52) during both sessions. This pattern may suggest that, in Experiment 2, participants changed their speed-accuracy ratio during the first session in favor of more speed, while sacrificing accuracy. Finally, in Experiment 3, neither speed (*L*) nor accuracy (*Q*) changed significantly during a test session.

In summary, our findings concerning changes in performance within a session with the FAIR-2 test are different from those reported for the d2 by Steinborn et al. (2018). These authors reported that performance in the d2 deteriorated within a session. It should be noted, however, that it is very difficult to interpret changes in performance within a test session when there are simultaneously strong performance changes between sessions. In particular, the strong performance improvements between sessions, as observed with complete repetitions, suggest that practice and learning have occurred during the first session, and it is quite likely to assume that practice and learning already improve performance during the first session. The fact that these improvements are not consistently observed during the first session strongly suggests, therefore, that these improvements are counter-acted by negative effects on performance induced by tiredness and/or loss of motivation.

## Conclusions

The fact that repeated testing with the FAIR-2 causes large and persistent improvements in test performance creates a problem for both the reliability and the validity of the test (e.g., Hagemeister & Westhoff, 2011; Schmidt-Atzert et al., 2004). Practice effects threaten the (retest) reliability of individual test results because the results of an individual may differ considerably between two sessions. In fact, in Experiment 1, individual performance could improve by up to 60% in the complete-repetition condition, but deteriorate by up to 40% in the role-reversal condition. Practice effects also threaten the validity of a test because it is unclear how much either ability or previous practice has contributed to a test result. These problems have implications for practical applications of the test and for future research. Concerning practical applications, the recommendation is to explore previous encounters with a particular test (Hagemeister & Westhoff, 2011; Schmidt-Atzert et al., 2004). If a participant does have previous experience with a particular test, then a different test should be used, because existing pen-and-paper tests of selective attention do not provide test norms for practiced individuals. If repeated testing is planned, for example, to assess the effectiveness of a treatment, two different versions of the FAIR-2 (to avoid ceiling effects in session 2) in combination with a control condition might be used. Concerning research, the present results suggest several lines for future research. First, future research could further explore the persistence of practice effects in the FAIR-2 and other pen-and-paper tests of visual selective attention. Second, such research could also assess the limits of practice: After how many repetitions do improvements stop occurring? Finally, future research might also further investigate the contents of stimulus-independent and stimulus-dependent learning in the FAIR-2 test.

## Supplementary Information

Below is the link to the electronic supplementary material.

Supplementary file1 (DOCX 43 KB)

Supplementary file2 (DOCX 25 KB)

## Data Availability

We had used pen-and-paper tests for collecting our raw data, and the resulting analog data cannot be made available on a public (digital) repository. However, we may provide Excel sheets with individual test scores upon request to the first author.

## Footnotes

1 The measure GZ-F is the sum of inspected items (i.e., targets plus distractors) minus errors. This measure was used for the d2, but is no longer used for the revised d2-R. In order to determine the sum of inspected items, participants were instructed to draw a vertical line behind the stimulus inspected last in a particular line when the tester gave the instruction “Stop. Next line”.

2 Rogers & Fisk, (1991) and Rogers (1992) compared the performance of younger and older participants, but here we confine our discussion to the results for the younger participants.

3 The target stimulus was a vertically bisected disc with a red half on the left and a green half on the right. The distractor stimuli were vertically bisected discs with a green half on the left and a red half on the right.

4 In the control condition, the target was a red horizontal bar on a green disc; the distractors either contained a green horizontal bar on a red disc or a vertical red bar on a green disc.

5 Steinborn et al. (2018) observed a very strong main effect of Session (${\eta }_{p}^{2}$ = .75). Similarly, Prinz (1979) observed a strong effect of condition (${\eta }_{p}^{2}$ = .66), which corresponds to the Condition × Session interaction observed in our study. Since we used different tests, we used a smaller estimation of effect size (i.e., ${\eta }_{p}^{2}$ = .20).

6 The Hans Huber Publishing Company, which holds the rights for the FAIR-2 test, generously provided written permission for us to create new variants of the FAIR test and to use these tests to investigate practice effects.
