# Supplementary material for: Effects of repeated testing in a pen-and-paper test of selective attention (FAIR-2)
Source: Psychol Res. 2021 Feb 11;86(1):294–311. doi: 10.1007/s00426-021-01481-x (PMC8821485; doi:10.1007/s00426-021-01481-x)
Supplement: Supplementary file 1 — Supplementary file1 (DOCX 43 KB) [file 426_2021_1481_MOESM1_ESM.docx]

**Appendix 1: Tables**

**Table 1.** Measures *L*, *Q*, and *K* as a function of the independent variables CONDITION, SESSION, and TEST PAGE observed in Experiment 1.

|  |  | Session 1 | |  | Session 2 | |
| --- | --- | --- | --- | --- | --- | --- |
|  |  | Test Page 1 | Test Page 2 |  | Test Page 1 | Test Page 2 |
| Measure *L* | Repetition | 204 | 212 |  | 263 | 262 |
|  | Role Reversal | 197 | 209 |  | 206 | 218 |
| Measure *Q* | Repetition | .943 | .933 |  | .982 | .973 |
|  | Role Reversal | .960 | .949 |  | .942 | .943 |
| Measure *K* | Repetition | 193 | 199 |  | 259 | 255 |
|  | Role Reversal | 190 | 199 |  | 195 | 207 |

**Note**. *L* = Sum of correctly marked targets; *Q* = percentage of correctly inspected items; *K* = *L* × *Q*.

**Table 2**. Results of three-factorial ANOVA for dependent variable *L* observed in Experiment 1.

| Source of Variance | df_num_ | df_den_ | MS_error_ | F | p | $\eta_{p}^{2}$ |
| --- | --- | --- | --- | --- | --- | --- |
| Condition | 1 | 47 | 5214 | 7.140 | .010 | .132 |
| Session | 1 | 47 | 1073 | 45.870 | < .001 | .494 |
| Test Page | 1 | 47 | 252 | 12.260 | .001 | .207 |
| Condition × Session | 1 | 47 | 1073 | 24.190 | < .001 | .340 |
| Condition × Test Page | 1 | 47 | 252 | 3.660 | .062 | .072 |
| Session × Test Page | 1 | 47 | 192 | 1.200 | .280 | .025 |
| Condition × Session × Test Page | 1 | 47 | 192 | 1.580 | .216 | .032 |

**Note**. num = numerator; den = denominator.

**Table 3**. Results of three-factorial ANOVA for dependent variable *Q* observed in Experiment 1.

| Source of Variance | df_num_ | df_den_ | MS_error_ | F | p | $\eta_{p}^{2}$ |
| --- | --- | --- | --- | --- | --- | --- |
| Condition | 1 | 47 | .003 | 1.480 | .230 | .031 |
| Session | 1 | 47 | .002 | 4.611 | .037 | .089 |
| Test Page | 1 | 47 | .003 | 3.273 | .077 | .065 |
| Condition × Session | 1 | 47 | .002 | 16.478 | < .001 | .260 |
| Condition × Test Page | 1 | 47 | < .001 | 0.267 | .608 | .006 |
| Session × Test Page | 1 | 47 | < .001 | 1.049 | .311 | .022 |
| Condition × Session × Test Page | 1 | 47 | < .001 | 0.743 | .393 | .016 |

**Note**. num = numerator; den = denominator.

**Table 4**. Results of three-factorial ANOVA for dependent variable *K* observed in Experiment 1.

| Source of Variance | df_num_ | df_den_ | MS_error_ | F | p | $\eta_{p}^{2}$ |
| --- | --- | --- | --- | --- | --- | --- |
| Condition | 1 | 47 | 5339 | 7.619 | .008 | .139 |
| Session | 1 | 47 | 1303 | 43.106 | < .001 | .478 |
| Test Page | 1 | 47 | 338 | 5.639 | .022 | .107 |
| Condition × Session | 1 | 47 | 1303 | 28.275 | < .001 | .376 |
| Condition × Test Page | 1 | 47 | 338 | 3.510 | .067 | .069 |
| Session × Test Page | 1 | 47 | 247 | 0.458 | .502 | .010 |
| Condition × Session × Test Page | 1 | 47 | 247 | 1.777 | .189 | .036 |

**Note**. num = numerator; den = denominator.

**Table 5**. Measures *L*, *Q*, and *K* as a function of the independent variables CONDITION, SESSION, and TEST PAGE observed in Experiment 2.

|  |  | Session 1 | |  | Session 2 | |
| --- | --- | --- | --- | --- | --- | --- |
|  |  | Test Page 1 | Test Page 2 |  | Test Page 1 | Test Page 2 |
| Measure *L* | Repetition | 180 | 187 |  | 238 | 233 |
|  | Role Reversal | 175 | 180 |  | 201 | 202 |
| Measure *Q* | Repetition | .964 | .946 |  | .970 | .963 |
|  | Role Reversal | .955 | .946 |  | .945 | .929 |
| Measure *K* | Repetition | 173 | 178 |  | 231 | 224 |
|  | Role Reversal | 167 | 171 |  | 190 | 188 |

**Note**. *L* = Sum of correctly marked targets; *Q* = percentage of correctly inspected items; *K* = *L* × *Q*.

**Table 6**. Results of three-factorial ANOVA for dependent variable *L* observed in Experiment 2.

| Source of Variance | df_num_ | df_den_ | MS_error_ | F | p | $\eta_{p}^{2}$ |
| --- | --- | --- | --- | --- | --- | --- |
| Condition | 1 | 55 | 5115 | 4.510 | .038 | .076 |
| Session | 1 | 55 | 855 | 95.304 | < .001 | .634 |
| Test Page | 1 | 55 | 305 | 0.908 | .345 | .016 |
| Condition × Session | 1 | 55 | 855 | 13.104 | < .001 | .192 |
| Condition × Test Page | 1 | 55 | 305 | 0.288 | .593 | .005 |
| Session × Test Page | 1 | 55 | 151 | 7.182 | .010 | .115 |
| Condition × Session × Test Page | 1 | 55 | 151 | 2.075 | .155 | .036 |

**Note**. num = numerator; den = denominator.

**Table 7**. Results of three-factorial ANOVA for dependent variable *Q* observed in Experiment 2.

| Source of Variance | df_num_ | df_den_ | MS_error_ | F | p | $\eta_{p}^{2}$ |
| --- | --- | --- | --- | --- | --- | --- |
| Condition | 1 | 55 | 0.004 | 4.490 | .039 | .075 |
| Session | 1 | 55 | 0.001 | 0.038 | .847 | .001 |
| Test Page | 1 | 55 | < 0.001 | 14.976 | < .001 | .214 |
| Condition × Session | 1 | 55 | 0.001 | 7.221 | .010 | .116 |
| Condition × Test Page | 1 | 55 | < 0.001 | 0.002 | .964 | < .001 |
| Session × Test Page | 1 | 55 | < 0.001 | 0.067 | .797 | .001 |
| Condition × Session × Test Page | 1 | 55 | < 0.001 | 1.499 | .226 | .027 |

**Note**. num = numerator; den = denominator.

**Table 8**. Results of three-factorial ANOVA for dependent variable *K* observed in Experiment 2.

| Source of Variance | df_num_ | df_den_ | MS_error_ | F | p | $\eta_{p}^{2}$ |
| --- | --- | --- | --- | --- | --- | --- |
| Condition | 1 | 55 | 4903 | 5.973 | .018 | .098 |
| Session | 1 | 55 | 1012 | 74.099 | < .001 | .574 |
| Test Page | 1 | 55 | 354 | 0.006 | .940 | < .001 |
| Condition × Session | 1 | 55 | 1012 | 13.645 | < .001 | .199 |
| Condition × Test Page | 1 | 55 | 354 | 0.205 | .652 | .004 |
| Session × Test Page | 1 | 55 | 171 | 6.531 | .013 | .106 |
| Condition × Session × Test Page | 1 | 55 | 171 | 0.803 | .374 | .014 |

**Note**. num = numerator; den = denominator.

**Table 9**. Features of Targets and Distractors in the eight different versions of the test used in Experiment 3.

The digits in brackets indicate the number of dots in each stimulus shape.

| Test | Targets | Distractors |
| --- | --- | --- |
| 1A | Triangle (1), Square (2) | Triangle (2), Square (1) |
| 1B | Triangle (2), Square (1) | Triangle (1), Square (2) |
| 2A | Triangle (3), Square (4) | Triangle (4), Square (3) |
| 2B | Triangle (4), Square (3) | Triangle (3), Square (4) |
| 3A | Circle (1), Diamond (2) | Circle (2), Diamond (1) |
| 3B | Circle (2), Diamond (1) | Circle (1), Diamond (2) |
| 4A | Circle (3), Diamond (4) | Circle (4), Diamond (3) |
| 4B | Circle (4), Diamond (3) | Circle (3), Diamond (4) |

**Table 10**. Measures *L*, *Q*, and *K* as a function of the independent variables CONDITION, SESSION, and TEST PAGE observed in Experiment 3.

|  |  | Session 1 | |  | Session 2 | |
| --- | --- | --- | --- | --- | --- | --- |
|  |  | Test Page 1 | Test Page 2 |  | Test Page 1 | Test Page 2 |
| Measure *L* | Repetition | 182 | 185 |  | 251 | 245 |
|  | Neutral | 185 | 192 |  | 208 | 208 |
|  | Role Reversal | 196 | 197 |  | 212 | 213 |
| Measure *Q* | Repetition | .948 | .940 |  | .977 | .964 |
|  | Neutral | .964 | .961 |  | .964 | .959 |
|  | Role Reversal | .945 | .943 |  | .940 | .938 |
| Measure *K* | Repetition | 172 | 173 |  | 245 | 237 |
|  | Neutral | 179 | 185 |  | 201 | 200 |
|  | Role Reversal | 185 | 186 |  | 202 | 202 |

**Note**. *L* = Sum of correctly marked targets; *Q* = percentage of correctly inspected items; *K* = *L* × *Q*.

**Table 11**. Results of three-factorial ANOVA for dependent variable *L* observed in Experiment 3.

| Source of Variance | df_num_ | df_den_ | MS_error_ | F | p | $\eta_{p}^{2}$ |
| --- | --- | --- | --- | --- | --- | --- |
| Condition | 2 | 65 | 5480 | 1.350 | .265 | .040 |
| Session | 1 | 65 | 950 | 80.546 | < .001 | .553 |
| Test Page | 1 | 65 | 244 | 0.365 | .548 | .006 |
| Condition × Session | 2 | 65 | 950 | 18.156 | < .001 | .358 |
| Condition × Test Page | 2 | 65 | 244 | 0.583 | .561 | .018 |
| Session × Test Page | 1 | 65 | 215 | 2.162 | .146 | .032 |
| Condition × Session × Test Page | 2 | 65 | 215 | 0.457 | .635 | .014 |

**Note**. num = numerator; den = denominator.

**Table 12**. Results of three-factorial ANOVA for dependent variable *Q* observed in Experiment 3.

| Source of Variance | df_num_ | df_den_ | MS_error_ | F | p | $\eta_{p}^{2}$ |
| --- | --- | --- | --- | --- | --- | --- |
| Condition | 2 | 65 | 0.005 | 2.240 | .115 | .064 |
| Session | 1 | 65 | 0.002 | 1.509 | .224 | .023 |
| Test Page | 1 | 65 | < 0.001 | 2.013 | .161 | .030 |
| Condition × Session | 2 | 65 | 0.002 | 3.183 | .048 | .089 |
| Condition × Test Page | 2 | 65 | < 0.001 | 0.464 | .631 | .014 |
| Session × Test Page | 1 | 65 | < 0.001 | 0.125 | .725 | .002 |
| Condition × Session × Test Page | 2 | 65 | < 0.001 | 0.067 | .935 | .002 |

**Note**. num = numerator; den = denominator.

**Table 13**. Results of three-factorial ANOVA for dependent variable *K* observed in Experiment 3.

| Source of Variance | df_num_ | df_den_ | MS_error_ | F | p | $\eta_{p}^{2}$ |
| --- | --- | --- | --- | --- | --- | --- |
| Condition | 2 | 65 | 5507 | 1.216 | .303 | .036 |
| Session | 1 | 65 | 1069 | 75.054 | < .001 | .536 |
| Test Page | 1 | 65 | 317 | 0.005 | .942 | < .001 |
| Condition × Session | 2 | 65 | 1069 | 18.666 | < .001 | .365 |
| Condition × Test Page | 2 | 65 | 317 | 0.735 | .483 | .022 |
| Session × Test Page | 1 | 65 | 317 | 1.802 | .184 | .027 |
| Condition × Session × Test Page | 2 | 65 | 317 | 0.325 | .723 | .010 |

**Note**. num = numerator; den = denominator.
