# Supplementary material for: Effects of repeated testing in a pen-and-paper test of selective attention (FAIR-2)
Source: Psychol Res. 2021 Feb 11;86(1):294–311. doi: 10.1007/s00426-021-01481-x (PMC8821485; doi:10.1007/s00426-021-01481-x)
Supplement: Supplementary file 2 — Supplementary file2 (DOCX 25 KB) [file 426_2021_1481_MOESM2_ESM.docx]

**Appendix 2: Combined Statistical Analysis of Experiments 1 and 2**

**Measure *L***. We first analyzed the impact of the time interval between two tests (Experiment 1: two weeks; Experiment 2: 13 weeks) on performance changes in *L*. Therefore, we computed *L* across both test pages for each participant and session, and subjected the scores to a three-factorial ANOVA with CONDITION (repetition vs. role reversal), SESSION (1 or 2), and EXPERIMENT (1 or 2) as independent variables. All three main effects were significant. The main effect of CONDITION reflected a higher *L* in the complete-repetition condition (*M* = 442, *SD* = 100) as compared to the role-reversal condition (*M* = 395, *SD* = 79), *F*(1, 102) = 11.598, *MSE* = 10,321, *p* < .001, $\eta_{p}^{2}$ = .102.The main effect of SESSION reflected an increase of *L* from the first session (*M* = 384, *SD* = 78) to the second session (*M* = 453, *SD* = 94), *F*(1, 102) = 133.24, *MSE* = 1911, *p* < .001, $\eta_{p}^{2}$ = .566. Finally, the main effect of EXPERIMENT reflected a higher L score in Experiment 1 (*M* = 441, *SD* = 92) than in Experiment 2 (*M* = 396, *SD* = 88), *F*(1, 102) = 9.982, *MSE* = 10,321, *p* = .002, $\eta_{p}^{2}$ = .089.

The two-way interaction of CONDITION × SESSION was the only-significant interaction, *F*(1, 102) = 37.820, *MSE* = 1911, *p* < .001, $\eta_{p}^{2}$ = .271. Wilcoxon tests revealed that *L* significantly increased from the first to the second session both for the complete-repetition condition (performance change = 106), *W* = 35.0, *p* < .001, *d* = -1.83, and for the role-reversal condition (performance change = 34), *W* = 322, *p* < .001, *d* = -0.516. Hence, the significant interaction resulted from a larger increase of *L* between sessions in the complete-repetition condition, as compared to the role-reversal condition.

The remaining *F* tests were not significant: EXPERIMENT × SESSION, *F*(1, 102) = 1.04, *p* = .310, $\eta_{p}^{2}$ = .010; CONDITION × EXPERIMENT, *F*(1, 102) = 0.281, *p* = .597, $\eta_{p}^{2}$ = .003; CONDITION × EXPERIMENT × SESSION, *F*(1, 102) = 2.22, *p* = .139, $\eta_{p}^{2}$ = .021.

**Measure *Q***. We also computed *Q* across both test pages for each participant and session, and subjected the scores to a three-factorial ANOVA with CONDITION (repetition vs. role reversal), SESSION (1 or 2), and EXPERIMENT (1 or 2) as independent variables. Only the main effect of CONDITION was significant. This main effect reflected a higher *Q* in the complete-repetition condition (*M* = .959, *SD* = .037) as compared to the role-reversal condition (*M* = .946, *SD* = .035), *F*(1, 102) = 5.513, *MSE* = 0.002, *p* = .021, $\eta_{p}^{2}$ = .051.The main effect of SESSION, *F*(1, 102) = 1.04, *p* = .310, $\eta_{p}^{2}$ = .010, and the main effect of EXPERIMENT, *F*(1, 102) = 0.015, *p* = .903, $\eta_{p}^{2}$ < .001, were not significant.

The two-way interaction of CONDITION × SESSION was the only-significant interaction, *F*(1, 102) = 24.05, *MSE* < 0.001, *p* < .001, $\eta_{p}^{2}$ = .191. Wilcoxon tests revealed that *Q* significantly increased from the first to the second session for the complete-repetition condition (performance change = .025), *W* = 258, *p* < .001, *d* = -0.550, whereas *Q* significantly decreased from the first to the second session for the role-reversal condition (performance change = -.012), *W* = 1033, *p* = .028, *d* = -0.348.

The *F* tests for the remaining interactions were not significant: EXPERIMENT × SESSION, *F*(1, 102) = 3.61, *p* = .060, $\eta_{p}^{2}$ = .005; CONDITION × EXPERIMENT, *F*(1, 102) = 0.534, *p* = .467, $\eta_{p}^{2}$ = .005; CONDITION × EXPERIMENT × SESSION, *F*(1, 102) = 2.92, *p* = .091, $\eta_{p}^{2}$ = .028.

**Measure *K***. Finally, we computed *K* across both test pages for each participant and session, and subjected the scores to a three-factorial ANOVA. Mirroring the results of the analysis of measure *L*, all three main effects were significant. The main effect of CONDITION reflected a higher *K* in the complete-repetition condition (*M* = 425, *SD* = 102) as compared to the role-reversal condition (*M* = 374, *SD* = 79), *F*(1, 102) = 13.679, *MSE* = 10,214, *p* < .001, $\eta_{p}^{2}$ = .118.The main effect of SESSION reflected an increase of *K* from the first session (*M* = 365, *SD* = 77) to the second session (*M* = 435, *SD* = 97), *F*(1, 102) = 112.686, *MSE* = 2,300, *p* < .001, $\eta_{p}^{2}$ = .525. Finally, the main effect of EXPERIMENT reflected a higher *K* score in Experiment 1 (*M* = 421, *SD* = 96) than in Experiment 2 (*M* = 378, *SD* = 88), *F*(1, 102) = 9.650, *MSE* = 10,214, *p* = .002, $\eta_{p}^{2}$ = .086.

The two-way interaction of CONDITION × SESSION was the only-significant interaction, *F*(1, 102) = 42.475, *MSE* < 2,300, *p* < .001, $\eta_{p}^{2}$ = .294. Wilcoxon tests revealed that *K* significantly increased from the first to the second session both for the complete-repetition condition (performance change = 113), *W* = 25.000, *p* < .001, *d* = 0.962, and for the role-reversal condition (performance change = 28), *W* = 421.000, *p* = .004, *d* = 0.453. Hence, the significant interaction resulted from a larger increase of *K* between sessions in the complete-repetition condition, as compared to the role-reversal condition.

The remaining *F* tests were not significant: EXPERIMENT × SESSION, *F*(1, 102) = 0.138, *p* = .711, $\eta_{p}^{2}$ = .001; CONDITION × EXPERIMENT, *F*(1, 102) = 0.191, *p* = .663, $\eta_{p}^{2}$ = .002; CONDITION × EXPERIMENT × SESSION, *F*(1, 102) = 3.198, *p* = .077, $\eta_{p}^{2}$ = .030.
